# Improving fiducial and prostate capsule visualization for radiotherapy planning using MRI

**DOI:** 10.1002/acm2.12529

**Published:** 2019-02-12

**Authors:** Angela U. Pathmanathan, Maria A. Schmidt, Douglas H. Brand, Evanthia Kousi, Nicholas J. van As, Alison C. Tree

**Affiliations:** ^1^ The Royal Marsden Hospital NHS Foundation Trust London UK; ^2^ The Institute of Cancer Research London UK

**Keywords:** geometric distortion, MR‐guided RT, MRI, prostate, segmentation

## Abstract

**Background and purpose:**

Intraprostatic fiducial markers (FM) improve the accuracy of radiotherapy (RT) delivery. Here we assess geometric integrity and contouring consistency using a T2*‐weighted (T2*W) sequence alone, which allows visualization of the FM.

**Material and methods:**

Ten patients scanned within the Prostate Advances in Comparative Evidence (PACE) trial (NCT01584258) had prostate images acquired with computed tomography (CT) and Magnetic Resonance (MR) Imaging: T2‐weighted (T2W) and T2*W sequences. The prostate was contoured independently on each imaging dataset by three clinicians. Interobserver variability was assessed using comparison indices with Monaco ADMIRE (research version 2.0, Elekta AB) and examined for statistical differences between imaging sets. CT and MR images of two test objects were acquired to assess geometric distortion and accuracy of marker positioning. The first was a linear test object comprising straight tubes in three orthogonal directions, the second was a smaller test object with markers suspended in gel.

**Results:**

Interobserver variability for prostate contouring was lower for both T2W and T2*W compared to CT, this was statistically significant when comparing CT and T2*W images. All markers are visible in T2*W images with 29/30 correctly identified, only 3/30 are visible in T2W images. Assessment of geometric distortion revealed in‐plane displacements were under 0.375 mm in MRI, and through plane displacements could not be detected. The signal loss in the MR images is symmetric in relation to the true marker position shown in CT images.

**Conclusion:**

Prostate T2*W images are geometrically accurate, and yield consistent prostate contours. This single sequence can be used to identify FM and for prostate delineation in a mixed MR‐CT workflow.

## 1 INTRODUCTION

Accurate co‐registration of magnetic resonance (MR) and computed tomography (CT) images is essential in radiotherapy (RT) planning using both modalities. MR‐CT fusion combines the superior soft tissue contrast of MR images and the electron density from CT images, which is currently required for planning.^1^ However, CT and MR examinations take place at different times and over different timescales; the acquisition of detailed MR images covering the tumor volume may require a few minutes, while CT is considerably faster. Physiological motion may thus affect MR and CT images differently, and this is detrimental to the accuracy of MR‐CT fusion. In addition, inter‐ and intra‐fraction motion may be significant at the time of RT delivery, introducing further errors.^2^, ^3^ In order to mitigate this, fiducial markers can be placed into relatively mobile tumors (or their vicinity), enabling more precise image co‐registration to be performed for MR‐CT fusion during the planning process^4^ and position verification prior to each fraction.^5^, ^6^ A more accurate MR‐CT co‐registration will enable better targeting, therefore markers must be visible, both in MR and CT.

Metallic markers appear bright on CT, often surrounded by reconstruction and beam hardening artifacts,^7^, ^8^ but do not yield MR signals and are seen as dark “void” areas on MR. Their susceptibility cause variations in the magnetic field in their vicinity, and they are often better visualized in T2*‐weighted (T2*W) images where the signal loss around the markers is emphasized.^9^ The design of MR protocols for RT planning thus requires not only geometric accuracy but also that the markers are clearly visible and the image contrast provides confidence in target outlining. Uncertainties and variation in target delineation during RT planning adds a further systematic error. MRI allows a reduction in interobserver variability for prostate contours compared to CT,^10^ however, this is dependent on the sequence used.^11^ Previously it has not been possible to provide one single sequence that enables both visualization of the markers and target outlining, and this adds a degree of complexity to the RT planning workflow.

This work investigates a sequence suitable for MR‐CT fusion for prostate RT using fiducial markers; in our institution, a set of three gold seeds is implanted in each patient. The MR protocol we implemented consists of two sequences; one standard T2‐weighted (T2W) sequence used in diagnostic prostate scans, thus optimized for visualization of intra‐prostatic structures, and a second T2*W sequence optimized for marker visualization using the combination of several gradient‐echoes with different echo‐times (TE) which follow each excitation. The second sequence maximizes visualization of the markers for RT planning fusion.

Studies so far for similar sequences have focused on accuracy of fiducial detection.^12^, ^13^, ^14^, ^15^, ^16^, ^17^ In this article we examine the T2*W sequence and investigate whether it is possible to use this sequence alone in prostate studies, considering geometric integrity, the ability to locate marker positions and the ability to provide enough contrast for prostate volume outlining.

## 2 MATERIALS AND METHODS

### 2.A Patient population

Patients were scanned at 1.5 T (Siemens Aera, Erlangen, Germany) as part of the Prostate Advances in Comparative Evidence (PACE) trial (NCT01584258). PACE A randomizes patients between prostatectomy and stereotactic body radiotherapy (SBRT) to a dose of 36.25 Gy in five fractions, and PACE B randomizes patients between SBRT and conventionally fractionated RT, either 62 Gy in 20 fractions or 78 Gy in 39 fractions. Patients do not receive androgen deprivation therapy. A minimum of 1 week prior to planning imaging, three 1.0 × 3.0 mm knurled gold markers are inserted into the prostate. Fiducial positions are used to fuse the CT and MR scans and for position verification prior to each treatment.

### 2.B Planning CT acquisition

At the Royal Marsden Hospital, all patients receiving RT in PACE have a RT planning CT followed, on the same day, by a planning MRI scan. Patients are scanned with bladder filling and rectal preparation as per institutional guidelines and no intravenous contrast is used. Patients receive 2 days of rectal preparation with enemas prior to planning, and an enema just before their planning CT scan. The CT scan incorporates axial slices of 1.5 mm from mid lumbar spine to below the obturator foramen.

### 2.C Planning MRI acquisition

Prostate MRI examinations were undertaken with two two‐dimensional (2D) sequences, covering the prostate volume in 28 adjacent slices (2.5 mm thickness). The first one is a standard T2W pulse sequence used in diagnostic MRI of the prostate. This sequence is based on fast spin‐echoes and allows visualization of internal structure of the prostate (central and peripheral zone and urethra). The second sequence is applied to the same locations, but it is gradient‐echo‐based and maximizes the signal loss surrounding the markers. For that purpose, we employed a sequence, which combines several gradient‐echo signals, with a range of echo‐times (TE), into one single image. This strategy maintains the signal‐to‐noise ratio in T2*W acquisitions and has been used for other clinical applications.^18^, ^19^ Both sequences cover the same volume, centered on the prostate and including at least part of the pelvic bones. Both sequences use the same shimming volume to optimize the magnetic field homogeneity and the manufacturer's own distortion correction software (in 2D). Parameters of both sequences are provided in Table 1.

**Table 1:** Parameters of MRI sequences for prostate RT Planning

	T2W acquisition (2D T2W FSE)	T2*W acquisition (2D “medic”)
FOV readout (phase)	240 mm (100%)	240 mm (100%)
PE oversampling	60%	60%
Number of Slices	28	28
Slice thickness/gap	2.5 mm/0	2.5 mm/0
Acquisition matrix (phase)	320 (75%)	256 (75%)
TE/TR	110 ms/7210 ms	24 ms/550 ms
Averages	3	2
Orientation	Transaxial	Transaxial
PE direction	Left/right	Left/right
Reconstruction matrix	320 × 320	512 × 512
Receiver bandwidth	200 Hz/pixel Fat‐water shift = 0.84 mm	230 Hz/pixel Fat‐water shift = 0.92 mm
Pixel size	0.75 mm × 0.75 mm	0.46875 mm × 0.46875 mm
Other	Echo‐train length 25, echo spacing 9.98 ms, echo‐trains per slice 16	Combined echoes 5, flip Angle 28 degrees
Filters	PrescanNormalize/DistCorrection 2D	PrescanNormalize/DistCorrection 2D
Coil arrangement	Spine coil & body array	Spine coil & body array
Total acquisition time	2 min 46 s Parallel imaging = 2 (GRAPPA)	6 min 4 s Parallel imaging = 2 (GRAPPA)

FSE: fast spin echo; FOV: field of view; TE: echo time; TR: relaxation time; GRAPPA: GeneRalized Autocalibrating Partial Parallel Acquisition.

### 2.D Geometric integrity

The field inhomogeneity of the main magnet and the non‐uniformity of gradient fields are known to progressively affect the MR images as the distance from the magnet isocenter increases. Although it is unlikely that the local MR‐CT co‐registration could be affected by geometric distortion at the prostate location, close to the isocenter, we characterized the hardware‐related geometric distortion over the imaging volume. For that purpose we acquired CT and MR images of a previously described test object consisting of straight tubes in three orthogonal directions, known as “Linear Test Object.”^20^ Images were co‐registered and evaluated using the three‐dimensional (3D) slicer software package (www.slicer.org).^21^ Displacements of test object structures between CT and MR images can be easily detected if they reach half of the voxel size — a level of accuracy that is sufficient for the purposes of this study.

In addition a second test object was built by suspending the markers in a gel volume comparable with a prostate (porcine gel, Sigma‐Aldricht, St. Louis, MI, 100 g/L, approximately 90 cm^3^) to verify whether the position of the markers is correctly depicted in the MR images with the sequences used. This step is necessary because the markers themselves disturb the field inhomogeneity, and the associated signal loss is not necessarily symmetric in relation to the true marker position.^22^ Therefore, in marker‐based registration, it is important to verify that systematic errors are not being introduced.

**Figure 1:**
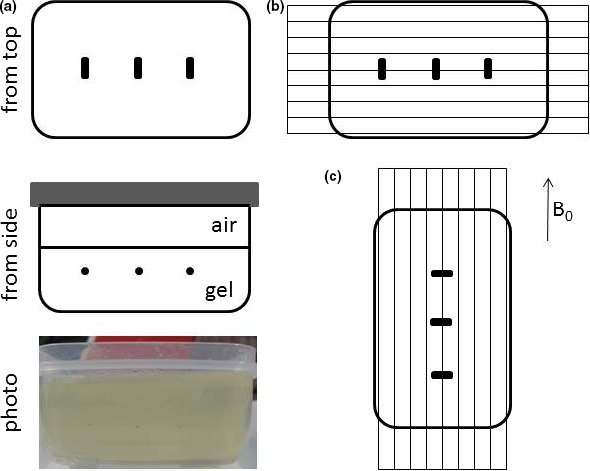
(a) Gel test object containing gold seeds (schematic diagram and photo showing gold seeds suspended in gel), (b) Standard test object position, gold seeds approximately lined up with main magnetic field as in most clinical examinations, and transaxial slices acquired, (c) Alternative orientation, gold seeds at 90° with static magnetic field B_0_. Images for slices A, B, and C are shown in Fig. 3.

The markers were orientated approximately in the superior/inferior direction, which most closely resembles their orientation in clinical examinations (Fig. 1). However, the object was rotated by 90° for a second MR acquisition, to evaluate how the susceptibility‐related signal loss depends on orientation, and also scanned at different orientations. In order to verify whether systematic errors were introduced, two CT‐MR registrations were produced. The first gold standard registration employs the outline of the test object volume, visible in MR and CT. The second registration employs only the marker information, and registration coordinates are compared. In addition, a capsule of cod liver oil was placed on top of the test object to provide a standard for displacements associated with chemical shift. The fat‐water chemical shift is known to be 3.5 ppm (225 Hz at 1.5 T), and fat‐water displacement was measured by using a readout gradient reversal.^23^

### 2.E Clinical studies

#### 2.E.1 Patient population

**Figure 2:**
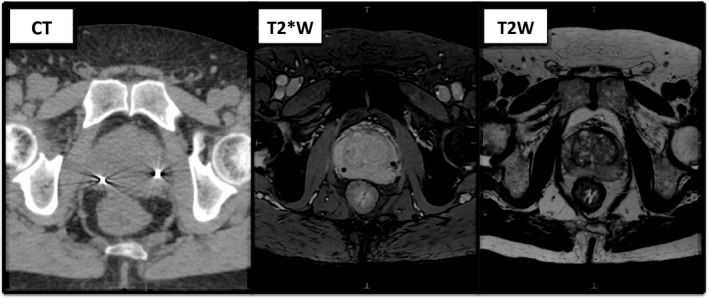
The three imaging sequences used for prostate contours showing the corresponding levels for the same patient. From left to right (a) CT imaging‐ fiducials seen as bright markers with surrounding artifact (b) T2*W MRI sequence‐ fiducials seen as dark void areas (c) T2W MRI sequence‐ fiducials not visible.

Ten patients with localized prostate cancer treated consecutively within the PACE trial with SBRT at the Royal Marsden Hospital, Sutton, from January 2015 to December 2016 were selected. Each patient had three imaging datasets‐ RT planning CT, T2W and T2*W MRI sequences as described. Examples are seen in Fig. 2.

#### 2.E.2 Visibility of fiducials

Without reference to the CT images, T2W and T2*W images were reviewed to assess the number of fiducial markers visible.

#### 2.E.3 Volume definition

Using Research Monaco 5.19.02 (Elekta AB, Stockholm, Sweden), the prostate contour was delineated on each of the three sequences for all ten patients by three clinicians from the same institution (AP, AT, and DB) experienced with prostate contouring on both CT and MRI. The clinicians were instructed to contour the prostate alone; that is, excluding the seminal vesicles (SV). Contouring was completed on each dataset independently, without reference to the other two types of imaging. The three sequences for each patient were contoured during three separate sessions, with at least 2 weeks between each session to minimize recall bias.

#### 2.E.4 Contour variability

Inter‐observer variability, as a measure of consistency, was assessed for each sequence by comparing each individual clinician contour to a Simultaneous Truth and Performance Level Estimation (STAPLE) contour^24^ formed from all three clinician contours.

Monaco ADMIRE software version 2.0 was used to generate a combination of contour comparison indices^25^, ^26^ to analyze the difference between clinician contours for the same imaging dataset. Distance measurements included the Hausdorff distance (HD) and mean distance between contours. Overlap measures included Dice similarity co‐efficient (DSC) and Cohen's Kappa. A shorter distance between contours or higher overlap index indicates higher agreement between observers. The Shapiro–Wilk test confirmed non‐normality of the data using SPSS Statistics, version 23. Therefore a separate Freidman's test was performed for all four delineation metrics, examining for differences across the three imaging modalities. Where significant, pair‐wise group comparison was undertaken using Wilcoxon's signed rank testing with Bonferroni correction.

## 3 RESULTS

### 3.A Geometric integrity

**Figure 3:**
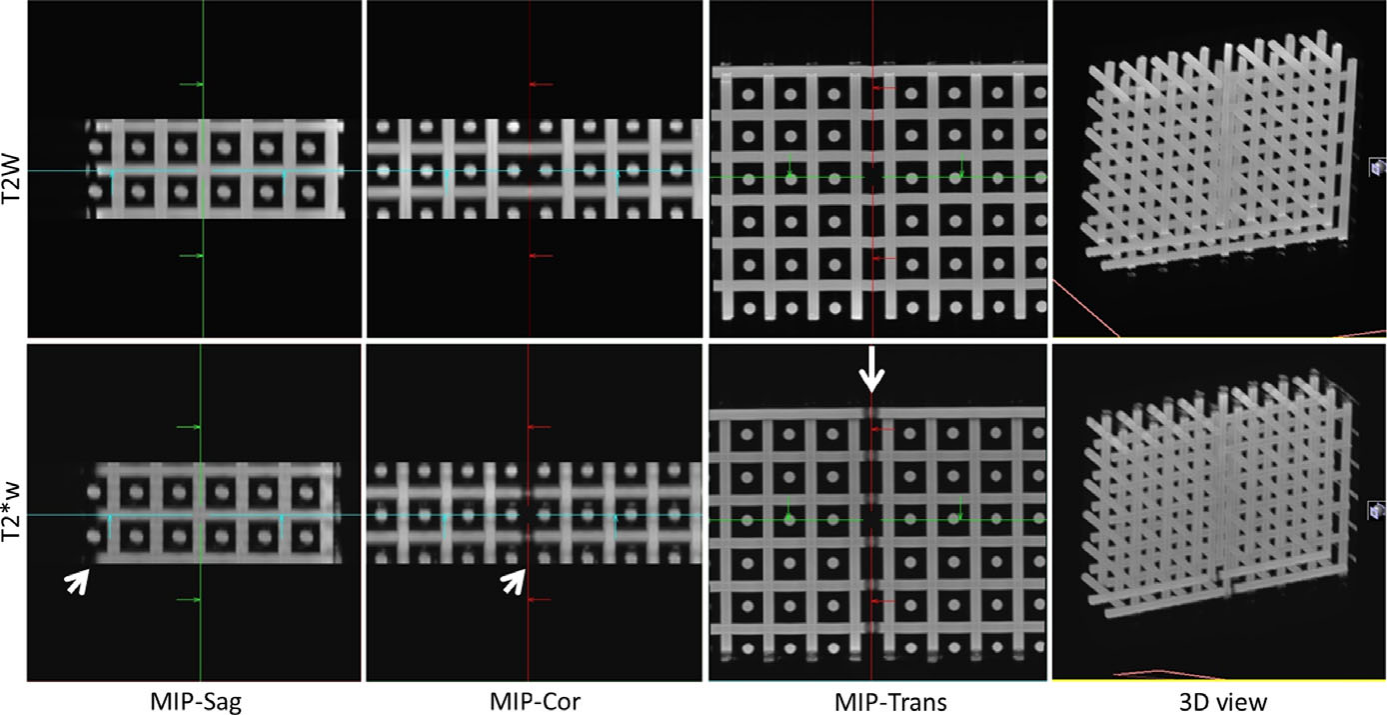
T2W (top) and T2*W (bottom) images of the Linear Test Object comprising straight tubes in three orthogonal directions. The maximum intensity projections (MIPs) show the brightest pixel along a given direction, in a three‐dimensional volume. All tubes appear straight (3D view) and overlap in the MIPs in all three directions. Signal loss associated with susceptibility‐related field inhomogeneity is visible in T2*W images (arrows), as expected.

Figure 3 shows Maximum Intensity Projections (MIPs) of the Linear Test Object dataset, and a 3D view for the T2W and T2*W sequences. All lines appear straight within the volume studied (240 × 240 × 70 mm^3^). Displacements from true position were estimated to be smaller than half of the voxel size (i.e., under 0.375 mm in the Left/Right and Anterior/Posterior direction). In the Superior/Inferior direction the slice thickness is 2.5 mm and no significant distortion could be detected. Using the T2*W sequences several imperfections of the test object become apparent as areas of signal loss associated with localized field inhomogeneity, but all tubes still appear straight.

**Figure 4:**
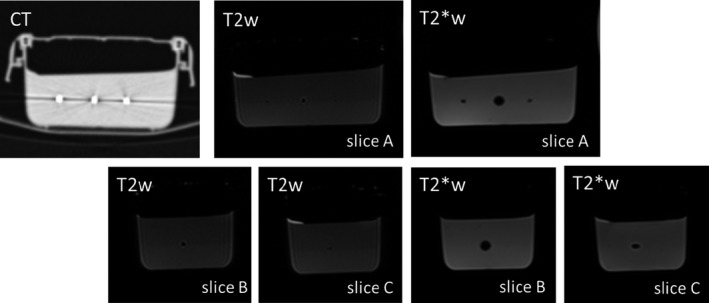
Gel test object images showing signal loss around marker positions, which is larger on T2*W images as expected. The signal loss is symmetric in relation to the true position of the marker. The level of signal loss associated with the markers varies, and is much larger for the central marker, irrespective of test object orientation. Ultrasound and CT images confirm there is no air gap or any imperfection at the markers. Image intensity differences within the gel in T2W images are due to the test object construction technique, in two layers; the second layer is built after the bottom layer has hardened sufficiently to hold the weight of the seeds.

Considering the test object with markers suspended in gel, the markers are always clearly visible in T2*W images; in T2W images the signal loss is much smaller, as expected (Fig. 4). MR and CT images were co‐registered and displacements were shown to be smaller than half pixel size. The signal loss in MR images was thus shown to be symmetric in relation to the true marker position shown in CT images. For both sequences the displacement of fat signals in relation to water signals due to chemical shift was confirmed to be less than 1 mm, as expected.

**Figure 5:**
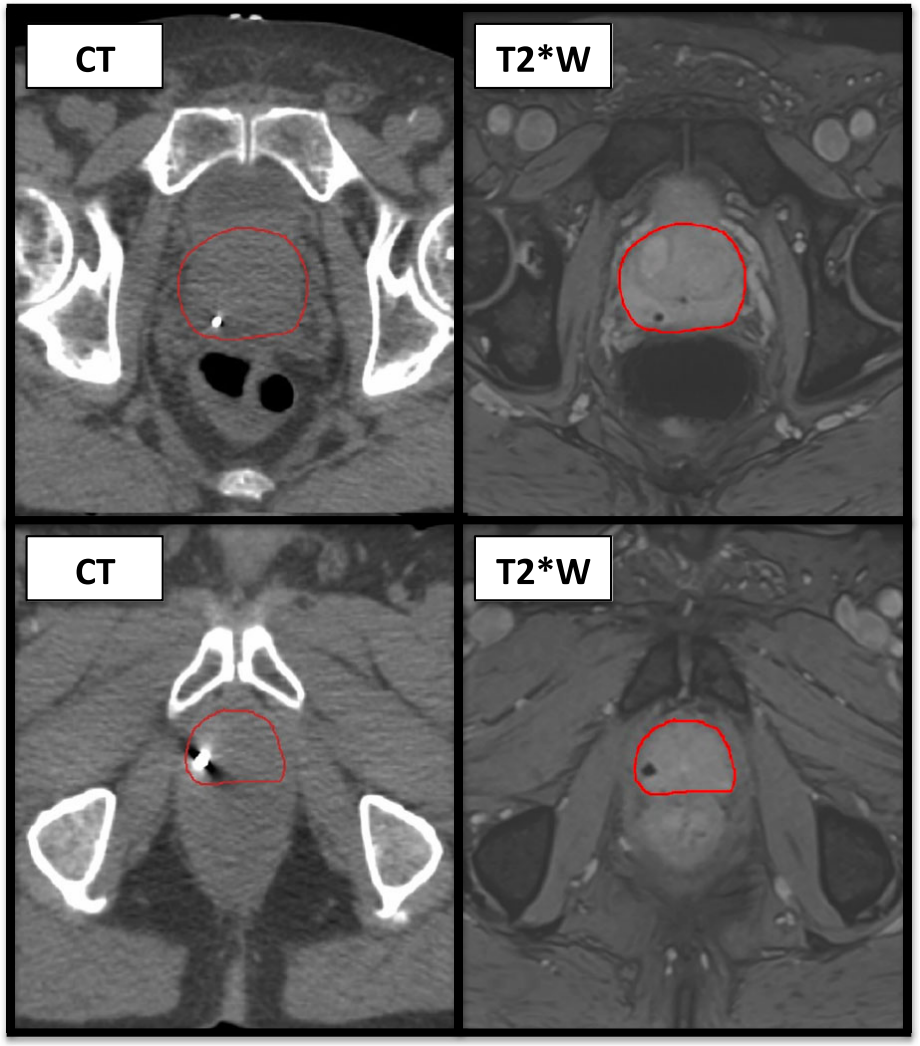
Clinical example of the variation in signal loss. Top line‐CT (left) and T2*W (right) imaging displaying the usual signal loss associated with a fiducial marker in the cranio‐caudal position. Bottom line‐CT (left) and T2*W (right) imaging for the same patient showing the altered signal loss seen with the inferior fiducial marker which in this case is angled more in the transverse plane.

Figure 5 shows an example of a clinical examination, with markers in different orientations. Both test object and clinical examinations show different levels of signal loss around the gold seeds.

A larger area of signal loss associated with the marker in the center of the gel test object was obtained irrespective of test object orientation, and was therefore investigated; the three markers appear identical in CT and ultrasound images and there are no visible air bubbles in the gel preparation. In order to gain further insight, the gel test object was rebuilt: the gold seeds were removed from the gel and cleaned with ethanol and placed in a new batch of gel in the same container, but in different positions. This resulted in almost identical images, the signal loss around one particular gold seed persisted being much larger than the signal loss surrounding the others, for any orientation. Therefore, although the signal loss pattern is expected to depend on seed orientation and position, it is also quite possible that one particular gold seed has a different magnetic susceptibility.

### 3.B Clinical studies

#### 3.B.1 Visibility of fiducials

**Figure 6:**
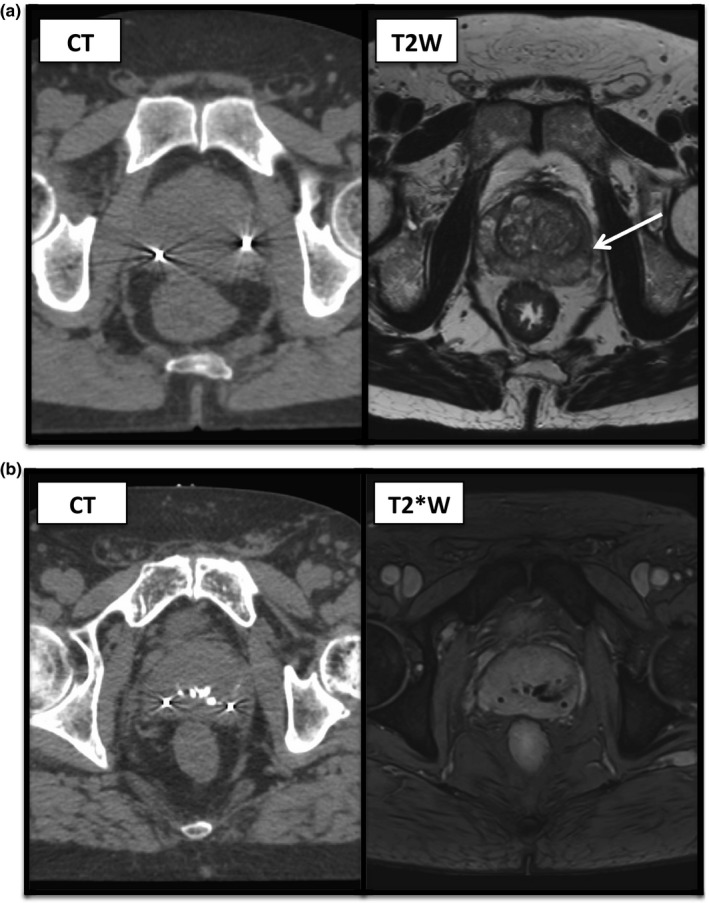
(a) Corresponding CT (left) and T2W (right) images for a patient showing the appearance of a fiducial marker on standard T2W imaging, as indicated by the arrow. The second fiducial marker visible on CT imaging could not be identified on T2W images here. (b) Corresponding CT (left) and T2*W (right) images for a patients showing two fiducials with surrounding artifact on CT images and central calcifications, all showing as signal loss on T2*W imaging.

Review of only the T2W imaging of all patients revealed three out of 30 fiducials were correctly identified. Fig. 6(a) shows an example of the fiducial appearance on T2W MRI. On T2*W imaging, all 30 fiducial markers were visible. However, only 29 out of 30 markers were correctly identified due to the presence of calcifications creating a similar signal loss. Such calcifications were variable in number and size but were seen in eight out of the ten patients, an example is seen in Fig. 6(b).

#### 3.B.2 Contour variability

Image review shows that the prostate has a high contrast appearance in relation to the surrounding tissues in T2*W images, and internal structures are not demonstrated as clearly as in T2W sequences. Summary of the comparison metrics for all ten patients for each imaging modality is seen in Table 2.

**Table 2:** Summary of the median comparison metrics for three observers contouring all ten patients for each imaging type (with interquartile range in brackets). * Denotes a statistically significant difference when compared to T2*W using a significance level of *P* = 0.0167 (Bonferroni correction)

Imaging modality	Hausdorff distance (mm)	Mean distance (mm)	Cohen's kappa	Dice similarity co‐efficient
CT	5.01* (4.68–5.71)	0.77* (0.69–0.86)	0.92* (0.89–0.93)	0.95* (0.94–0.96)
T2W	4.09 (3.57–4.89)	0.53 (0.48–0.61)	0.94 (0.93–0.96)	0.97 (0.96–0.97)
T2*W	3.61 (3.16–3.73)	0.45 (0.43–0.48)	0.95 (0.94–0.96)	0.97 (0.96–0.97)

There is good agreement between the three observers for all imaging modalities. Distance measurements between contours were greater and overlap indices lower for CT compared to both MR sequences, indicating a poorer interobserver variability for CT imaging compared to MRI. This was statistically significant when comparing CT with T2*W, as indicated in Table 2.

## 4 DISCUSSION

Test object images demonstrated that prostate MR images are not significantly distorted, and that the T2*W sequence produces a signal void that is symmetric in relation to the true marker position. This indicates that the signal loss is sufficiently large to obscure the volume immediately adjacent to the seeds where significant image distortion could otherwise be detected.^15^ Detected differences in the size of the signal void associated with markers are expected to relate to the marker orientation in relation to the static magnetic field and transaxial image plane,^22^, ^27^ but small variations in the magnetic susceptibility of the seeds cannot be ruled out as a contributing factor.

There is a high agreement for prostate contouring on all image sets, likely to reflect the high level of experience of all clinicians, from the same institution and familiar with using MRI for contouring. The higher agreement for contours on MRI compared to CT is consistent with previous studies as a result of the improved soft tissue contrast with MRI.^28^, ^29^ Despite the visual appearance of a more defined prostate capsule on the T2*W sequence, there was no significant difference in interobserver variability when compared to T2W imaging, which again may reflect the users’ experience with MR sequences. For this group of observers, the T2*W sequence is similar to standard T2W imaging, but with the added benefit of fiducial identification.

The more recent development of MR‐guided RT allows the use of continuous MRI during treatment for motion monitoring and gating.^30^ Ultimately the aim would be for an MR‐only workflow^31^ without the need for markers, using soft tissue visualization alone. In this context the T2*W sequence may be advantageous in comparison to the standard diagnostic T2W sequence as the prostate has a high intensity appearance and fewer internal structures are clearly depicted. The performance of automated contouring software based on machine learning and artificial intelligence techniques should therefore be investigated for the T2*W sequence. However, at present, MR‐guided delivery mostly relies on a mixed MR‐CT workflow with fiducials allowing more accurate fusion of images^4^ and further used for position verification prior to treatment.

There have been a number of studies investigating dedicated MRI sequences for fiducial detection.^12^, ^13^, ^14^, ^15^, ^16^, ^17^ Both balanced steady‐state free precession sequences^13^ and sequences based on spoiled gradient‐echoes have been employed in 2D^12^, ^13^, ^14^, ^15^ and 3D^16^, ^17^ acquisitions, relying on T2*‐related signal loss to create a detectable signal void in the vicinity of the fiducials. The averaging of consecutive echoes in multi‐echo recalled sequences, such as the one used here is an attractive mechanism to increase the signal‐to‐noise ratio. Previous investigations of pulse sequences of this type focused on seed depiction capabilities; Shieda et al.^12^ report superior image sharpness, but did not perform contouring studies. We demonstrated a successful combination of prostate contouring and correct seed localization with one single sequence. Furthermore, we demonstrated the absence of geometric distortions which could lead to systematic registration errors. We believe this is a valuable advance toward MR‐only prostate RT planning.

The accuracy of fiducial detection is paramount and can be either manual^12^ or automatic.^13^, ^14^, ^15^, ^16^, ^17^ However, ultimately, this must be performed automatically, especially if intrafractional imaging is to be used. Different methods have been described for automatic algorithms including feature extraction^13^, ^15^ and template matching.^14^, ^16^, ^17^ The fiducial detection is dependent on the signal loss, which varies with factors including seed orientation and TE.^22^, ^27^ We demonstrated that calcifications in prostate are a common source of signal voids in T2*W images, and they have been shown to mimic fiducial voids.^32^ Although Gustafsson et al.^15^ proposed to detect fiducials automatically by considering images at different TEs and the progressive increase in signal loss in multiple‐echo pulse sequences, it is unclear whether calcifications will be a significant confounding factor. Further investigation is required to determine whether false positive detection as a result of calcifications is a significant issue and whether calcifications can contribute towards MR‐CT co‐registration.^32^ The full potential of artificial intelligence techniques in fiducial detection has not yet been realized.^33^

With progressively more targeted treatment delivery, the accuracy of delineation becomes even more essential.^34^ For the prostate, this requires adequate tissue contrast of the capsule to improve confidence in contouring and reduce inter‐observer variability. With the development of prostate motion monitoring in MR‐guided RT, the prostate contour can be used for gated treatment.^35^ This requires easy and accurate identification of the target either visually or using automated algorithms. The latter may either rely on registration of images and propagation of contours or *de novo* auto‐delineation of the prostate on new images.^36^, ^37^, ^38^ The sequence described here would therefore be an attractive solution for detailing seeds and the prostate capsule. Further work of significance to MR‐guided RT, will be assessment of prostate contouring by treatment radiographers^39^ and auto‐contouring software on the sequences used here.

## 5 CONCLUSION

We have described here a single T2*W MR sequence suitable for fiducial depiction and prostate contouring. These MR images were demonstrated to be geometrically accurate, the MR signal loss surrounding the fiducial was shown to be symmetric in relation to the true marker position shown in CT and all markers are visible. Prostate contours on MR are more consistent than CT‐based contours with good agreement between prostate RT clinicians. We expect T2*W sequences to be useful for a mixed MR‐CT workflow and furthermore for MR‐guided RT.

## CONFLICTS AND DISCLOSURE

The Royal Marsden Hospital NHS Foundation Trust and Institute of Cancer Research are part of the Elekta MR‐Linac research consortium, which aims to coordinate international research into the MR‐Linac. Elekta and Philips are members of the MR‐Linac Consortium. Elekta financially supports the MR‐linac Consortium and all member institutes, including research funding. AT and AP have received research and educational travel support from Elekta. Elekta supports travel costs for consortium meetings. AT has received honoraria from Janssen, Astellas, Ferring and Bayer and research funding from MSD outside of the submitted work.

## References

[1] Schmidt AM , Payne SG . Radiotherapy planning using MRI. Phys Med Biol. 2015;60:R323. 26509844 10.1088/0031-9155/60/22/R323 PMC5137785

[2] Langen KM , Jones DTL . Organ motion and its management. Int J Radiat Oncol Biol Phys. 2001;50:265–278. 11316572 10.1016/s0360-3016(01)01453-5

[3] McPartlin AJ , Li XA , Kershaw LE , et al. MRI‐guided prostate adaptive radiotherapy – a systematic review. Radiother Oncol. 2016;119:371–380. 27162159 10.1016/j.radonc.2016.04.014

[4] Parker CC , Damyanovich A , Haycocks T , Haider M , Bayley A , Catton CN . Magnetic resonance imaging in the radiation treatment planning of localized prostate cancer using intra‐prostatic fiducial markers for computed tomography co‐registration. Radiother Oncol. 2003;66:217–224. 12648794 10.1016/s0167-8140(02)00407-3

[5] van der Heide UA , Kotte AN , Dehnad H , Hofman P , Lagenijk JJ , van Vulpen M . Analysis of fiducial marker‐based position verification in the external beam radiotherapy of patients with prostate cancer. Radiother Oncol. 2007;82:38–45. 17141903 10.1016/j.radonc.2006.11.002

[6] Beltran C , Herman MG , Davis BJ . Planning target margin calculations for prostate radiotherapy based on intrafraction and interfraction motion using four localization methods. Int J Radiat Oncol Biol Phys. 2008;70:289–295. 17919837 10.1016/j.ijrobp.2007.08.040

[7] Meyer E , Raupach R , Lell M , Schmidt B , Kachelriess M . Normalized metal artifact reduction (NMAR) in computed tomography. Med Phys. 2010;37:5482–5493. 21089784 10.1118/1.3484090

[8] Boas FE , Fleischmann D . Evaluation of two iterative techniques for reducing metal artifacts in computed tomography. Radiology. 2011;259:894–902. 21357521 10.1148/radiol.11101782

[9] Callaghan PT . Principles of Nuclear Magnetic Resonance Microscopy. Oxford: Clarendon Press; 1991:208–217.

[10] Rasch C , Barillot I , Remeijer P , Touw A , van Herk M , Lebesque JV . Definition of the prostate in CT and MRI: a multi‐observer study. Int J Radiat Oncol Biol Phys. 1999;43:57–66. 9989514 10.1016/s0360-3016(98)00351-4

[11] Nyholm T , Jonsson J , Söderström K , et al. Variability in prostate and seminal vesicle delineations defined on magnetic resonance images, a multi‐observer, ‐center and ‐sequence study. Radiat Oncol (London, England). 2013;8:126. 10.1186/1748-717X-8-126 PMC3680182 23706145

[12] Schieda N , Avruch L , Shabana WM , Malone SC . Multi‐echo gradient recalled echo imaging of the pelvis for improved depiction of brachytherapy seeds and fiducial markers facilitating radiotherapy planning and treatment of prostatic carcinoma. J Magnet Reson Imaging. 2015;41:715–720. 10.1002/jmri.24590 24510444

[13] Dinis Fernandes C , Dinh CV , Steggerda MJ , et al. Prostate fiducial marker detection with the use of multi‐parametric magnetic resonance imaging. Phys Imaging Radiat Oncol. 2017;1:14–20.

[14] Ghose S , Mitra J , Rivest‐Henault D , et al. MRI‐alone radiation therapy planning for prostate cancer: automatic fiducial marker detection. Med Phys. 2016;43:2218. 27147334 10.1118/1.4944871

[15] Gustafsson C , Korhonen J , Persson E , Gunnlaugsson A , Nyholm T , Olsson LE . Registration free automatic identification of gold fiducial markers in MRI target delineation images for prostate radiotherapy. Med Phys. 2017;44:5563–5574. 28803447 10.1002/mp.12516

[16] Zijlstra F , Moerland MA , van der Voort van Zyp JRN , Noteboom JL , Viergever MA , Seevinck PR . Challenges in MR‐only seed localization for postimplant dosimetry in permanent prostate brachytherapy. Med Phys. 2017;44:5051–5060. 28777451 10.1002/mp.12505

[17] Maspero M , van den Berg CAT , Zijlstra F , et al. Evaluation of an automatic MR‐based gold fiducial marker localisation method for MR‐only prostate radiotherapy. Phys Med Biol. 2017;62:7981–8002. 28825917 10.1088/1361-6560/aa875f

[18] Martin N , Malfair D , Zhao Y , et al. Comparison of MERGE and axial T2‐weighted fast spin‐echo sequences for detection of multiple sclerosis lesions in the cervical spinal cord. AJR Am J Roentgenol. 2012;199:157–162. 22733907 10.2214/AJR.11.7039

[19] Held P , Dorenbeck U , Seitz J , Frund R , Albrich H . MRI of the abnormal cervical spinal cord using 2D spoiled gradient echo multiecho sequence (MEDIC) with magnetization transfer saturation pulse. A T2* weighted feasibility study. J Neuroradiol (Journal de neuroradiologie). 2003;30:83–90. 12717293

[20] Doran SJ , Charles‐Edwards L , Reinsberg SA , Leach MO . A complete distortion correction for MR images: I. Gradient warp correction. Phys Med Biol. 2005;50:1343–1361. 15798328 10.1088/0031-9155/50/7/001

[21] Fedorov A , Beichel R , Kalpathy‐Cramer J , et al. 3D Slicer as an image computing platform for the quantitative imaging network. Magn Reson Imaging. 2012;30:1323–1341. 22770690 10.1016/j.mri.2012.05.001 PMC3466397

[22] Jonsson JH , Garpebring A , Karlsson MG , Nyholm T . Internal fiducial markers and susceptibility effects in MRI‐simulation and measurement of spatial accuracy. Int J Radiat Oncol Biol Phys. 2012;82:1612–1618. 21477942 10.1016/j.ijrobp.2011.01.046

[23] Chang H , Fitzpatrick JM . A technique for accurate magnetic resonance imaging in the presence of field inhomogeneities. IEEE Trans Med Imaging. 1992;11:319–329. 18222873 10.1109/42.158935

[24] Warfield SK , Zou KH , Wells WM . Simultaneous truth and performance level estimation (STAPLE): an algorithm for the validation of image segmentation. IEEE Trans Med Imaging. 2004;23:903–921. 15250643 10.1109/TMI.2004.828354 PMC1283110

[25] Hanna GG , Hounsell AR , O'Sullivan JM . Geometrical analysis of radiotherapy target volume delineation: a systematic review of reported comparison methods. Clin Oncol (Royal College of Radiologists (Great Britain)). 2010;22:515–525. 10.1016/j.clon.2010.05.006 20554168

[26] Fotina I , Lutgendorf‐Caucig C , Stock M , Potter R , Georg D . Critical discussion of evaluation parameters for inter‐observer variability in target definition for radiation therapy. Strahlenther Onkol. 2012;188:160–167. 22281878 10.1007/s00066-011-0027-6

[27] Schenck JF . The role of magnetic susceptibility in magnetic resonance imaging: MRI magnetic compatibility of the first and second kinds. Med Phys. 1996;23:815–80. 8798169 10.1118/1.597854

[28] Villeirs GM , Vaerenbergh K , Vakaet L , et al. Interobserver delineation variation using CT versus combined CT + MRI in intensity–modulated radiotherapy for prostate cancer. Strahlenther Onkol. 2005;181:424–430. 15995835 10.1007/s00066-005-1383-x

[29] Debois M , Oyen R , Maes F , et al. The contribution of magnetic resonance imaging to the three‐dimensional treatment planning of localized prostate cancer. Int J Radiat Oncol Biol Phys. 1999;45:857–865. 10571190 10.1016/s0360-3016(99)00288-6

[30] Pathmanathan AU , van As NJ , Kerkmeijer LGW , et al. Magnetic resonance imaging‐guided adaptive radiation therapy: a “game changer” for prostate treatment? Int J Radiat Oncol Biol Phys. 2018;100:361–373. 29353654 10.1016/j.ijrobp.2017.10.020

[31] Nyholm T , Jonsson J . Counterpoint: opportunities and challenges of a magnetic resonance imaging–only radiotherapy work flow. Sem Radiat Oncol. 2014;24:175–180. 10.1016/j.semradonc.2014.02.005 24931088

[32] Zeng GG , McGowan TS , Larsen TM , et al. Calcifications are potential surrogates for prostate localization in image‐guided radiotherapy. Int J Radiat Oncol Biol Phys. 2008;72:963–966. 18954708 10.1016/j.ijrobp.2008.07.021

[33] LeCun Y , Bengio Y , Hinton G . Deep learning. Nature. 2015;521:436–444. 26017442 10.1038/nature14539

[34] Njeh C . Tumor delineation: the weakest link in the search for accuracy in radiotherapy. J Med Phys. 2008;33:136–140. 19893706 10.4103/0971-6203.44472 PMC2772050

[35] Bohoudi O , Bruynzeel A , Senan S , Slotman B , Palacios M , Lagerwaard F . Using a MRI‐guided radiation therapy system for prostate cancer patients. ESTRO 36; 2017:SP‐0494.

[36] Greenham S , Dean J , Fu CKK , et al. Evaluation of atlas‐based auto‐segmentation software in prostate cancer patients. J Med Radiat Sci. 2014;61:151–158. 26229651 10.1002/jmrs.64 PMC4175851

[37] Klein S , van der Heide UA , Lips IM , van Vulpen M , Staring M , Pluim JPW . Automatic segmentation of the prostate in 3D MR images by atlas matching using localized mutual information. Med Phys. 2008;35:1407–1417. 18491536 10.1118/1.2842076

[38] Pasquier D , Lacornerie T , Vermandel M , Rousseau J , Lartigau E , Betrouni N . Automatic segmentation of pelvic structures from magnetic resonance images for prostate cancer radiotherapy. Int J Radiat Oncol Biol Phys. 2007;68:592–600. 17498571 10.1016/j.ijrobp.2007.02.005

[39] Pathmanathan AU , McNair HA , Schmidt MA , et al. Comparison of prostate delineation on multimodality imaging for MR‐guided radiotherapy. Br J Radiol. 2019;92:20180948. 30676772 10.1259/bjr.20180948 PMC6540870

